# Risk factors for pneumonia and influenza hospitalizations in long-term care facility residents: a retrospective cohort study

**DOI:** 10.1186/s12877-020-1457-8

**Published:** 2020-02-10

**Authors:** Patience Moyo, Andrew R. Zullo, Kevin W. McConeghy, Elliott Bosco, Robertus van Aalst, Ayman Chit, Stefan Gravenstein

**Affiliations:** 1 Department of Health Services, Policy, and Practice, Brown University School of Public Health, 121 South Main Street, Box G-S121-6, Providence, RI 02912 USA; 2 Center for Gerontology and Health Care Research, School of Public Health, Brown University School of Public Health, Providence, RI USA; 3 Department of Epidemiology, Brown University School of Public Health, Providence, RI USA; 4 Center of Innovation in Long-Term Services and Supports, Providence Veterans Affairs Medical Center, Providence, RI USA; 5 Sanofi Pasteur, Swiftwater, PA USA; 6 Department of Health Sciences, University of Groningen, University Medical Center Groningen, Groningen, the Netherlands; 7 Leslie Dan School of Pharmacy, University of Toronto, Toronto, Ontario Canada

**Keywords:** Pneumonia, Influenza, Medicare, Long-term care, Nursing homes

## Abstract

**Background:**

Older adults who reside in long-term care facilities (LTCFs) are at particularly high risk for infection, morbidity and mortality from pneumonia and influenza (P&I) compared to individuals of younger age and those living outside institutional settings. The risk factors for P&I hospitalizations that are specific to LTCFs remain poorly understood. Our objective was to evaluate the incidence of P&I hospitalization and associated person- and facility-level factors among post-acute (short-stay) and long-term (long-stay) care residents residing in LTCFs from 2013 to 2015.

**Methods:**

In this retrospective cohort study, we used Medicare administrative claims linked to Minimum Data Set and LTCF-level data to identify short-stay (< 100 days, index = admission date) and long-stay (100+ days, index = day 100) residents who were followed from the index date until the first of hospitalization, LTCF discharge, Medicare disenrollment, or death. We measured incidence rates (IRs) for P&I hospitalization per 100,000 person-days, and estimated associations with baseline demographics, geriatric syndromes, clinical characteristics, and medication use using Cox regression models.

**Results:**

We analyzed data from 1,118,054 short-stay and 593,443 long-stay residents. The crude 30-day IRs (95% CI) of hospitalizations with P&I in the principal position were 26.0 (25.4, 26.6) and 34.5 (33.6, 35.4) among short- and long-stay residents, respectively. The variables associated with P&I varied between short and long-stay residents, and common risk factors included: advanced age (85+ years), admission from an acute hospital, select cardiovascular and respiratory conditions, impaired functional status, and receipt of antibiotics or Beers criteria medications. Facility staffing and care quality measures were important risk factors among long-stay residents but not in short-stay residents.

**Conclusions:**

Short-stay residents had lower crude 30- and 90-day incidence rates of P&I hospitalizations than long-stay LTCF residents. Differences in risk factors for P&I between short- and long-stay populations suggest the importance of considering distinct profiles of post-acute and long-term care residents in infection prevention and control strategies in LTCFs. These findings can help clinicians target interventions to subgroups of LTCF residents at highest P&I risk.

## Background

Older adults (≥65 years of age) in long-term care facilities (LTCFs) have a high risk of infection, hospitalization, and death due to respiratory infections such as pneumonia and influenza (P&I) [1–4]. These infections contribute to a substantial share of transfers to acute care hospitals [5], with nearly one third of LTCF residents with pneumonia who may require hospital admission [6]. Despite the significant morbidity and economic burden imposed by P&I among older adults, including in non-epidemic years [7–9], there is scant contemporary research that comprehensively assesses the risk factors for P&I resulting in hospitalization among LTCF residents. Particular focus on P&I among LTCF residents, whether short- or long-stay, is especially warranted given that close living quarters and shared caregivers found in an institutional environment can increase the risk of exposure to infections and rate of transmission [10].

Prior research focused on P&I hospitalization among LTCF residents has also been limited in the number and geographic distribution of facilities examined [5, 6, 11], and most existing studies overlook the distinction between short- and long-stay LTCF residents in their analyses [12–14]. This is a relevant clinical and research consideration given rising numbers of short-stay (i.e., post-acute care) residents and distinct care goals and needs that distinguish them from long-stay residents [15–17]. For example, short-stay residents typically require rehabilitative nursing immediately following hospitalization, whereas long-stay residents predominantly receive custodial and chronic care services [18].

This study builds on a previous research that found potentially modifiable facility characteristics including greater workforce hiring, more staffing hours, and higher quality care practices were associated with lower incidence rates for P&I hospitalization among LTCF residents [19]. To our knowledge, the Bosco et al. paper is the only one on the topic to consider short- and long-stay LTCF residents separately in its analyses. While this prior study provides an understanding of the facility-level structural and operational targets for improving infection control and prevention in LTCFs, there remain knowledge gaps on the individual-level risk factors and epidemiology of P&I infections among older adults in LTCFs. Other studies report variable respiratory infection incidence estimates ranging from 1.1 to 85.2%, often without examining risk factors. In the limited instances where examined, individual-level risk factors for P&I including difficulty with swallowing and lack of influenza vaccination have been reported [6]. A study of LTCF residents in Japan identified reduced activities of daily living status, swallowing dysfunction, under-nourishment, ischemic heart disease, and dementia as risk factors for incident pneumonia [20].

We investigated P&I events in a national sample of Medicare beneficiaries residing in LTCFs from 2013 to 2015. Our objectives were to 1) determine the incidence of P&I hospitalizations among short- and long-stay residents, and 2) assess individual and facility-level risk factors for P&I hospitalizations. We hypothesized that resident-level demographics, medical conditions, geriatric syndromes, and medication use, and facility-level characteristics including staffing and care quality would be associated with P&I.

## Methods

### Study design and data sources

This was a retrospective cohort study using Medicare enrollment, and Parts A and D claims linked to Minimum Data Set (MDS) for 100% of LTCF residents enrolled in fee-for-service Medicare during 2013–2015. Medicare Part A data were used to identify hospitalizations involving P&I, and Part D claims enabled the ascertainment of prescribed medications. The MDS is a federally required clinical assessment completed at admission and at least quarterly thereafter among all residents in Medicare or Medicaid certified nursing homes. MDS data provide a comprehensive and standardized assessment of the functional capabilities and health needs of LTCF residents [21, 22]. Specifically, MDS data include demographics, clinical conditions, treatments, behaviors, physical function, and cognitive status. We applied the residential history file algorithm to track the timing and location of health services utilization [23]. Facility-level variables were obtained from Online Survey and Certification And Survey Provider Enhanced Reports (OSCAR/CASPER) and LTCFocus data collected for all Medicare- and Medicaid-certified LTCFs. This study was approved by the Brown University Institutional Review Board.

### Study population

The study cohort was derived from a national source population of Medicare beneficiaries residing in LTCFs between January 1, 2013 and December 31, 2015. Eligible residents were categorized as short-stay (total stay < 100 days in the same LTCF), or long-stay (total stay ≥100 consecutive days with ≤10 days outside the facility). Index dates were defined as the LTCF admission date for short-stay residents and day 100 of a stay for long-stay residents. We sampled the first LTCF stay, and followed residents from their respective index dates until hospitalization, discharge from the LTCF, disenrollment from Medicare, death, or end of the study period, whichever occurred first. The cohort inclusion criteria were 1) continuous enrollment in Medicare Parts A and D 6 months prior to index; 2) age at index ≥65 years; and 3) ≥1 MDS assessment within 100 days before the index date for long-stay residents and upon entry to the facility for short-stay residents. We excluded residents with Medicare Advantage enrollment, who received hospice services, or had missing data on any covariate used in analyses.

### Resident-level risk factors

Risk factors were selected based on prior literature and our clinical experience related to what factors could influence P&I risk [6, 19, 24–26]. Resident characteristics were measured during the 6-month period prior to, or at, the index date to ensure they were not influenced by the outcome. We evaluated demographic, tobacco use, body mass index, clinical (diagnoses and geriatric syndromes), medication use and health service use variables as potential risk factors for P&I hospitalizations. Demographic factors included age, sex, race and ethnicity. Clinical diagnoses from MDS included, e.g., cancer, atrial fibrillation, history of pneumonia, diabetes mellitus, arthritis, Alzheimer’s disease, asthma/chronic obstructive pulmonary disease (COPD)/chronic lung disease. Among others [27], geriatric syndromes included, e.g., cognitive function scale score [28], Changes in Health, End-stage disease and Symptoms and Signs (CHESS) scale score [29], and activities of daily living (ADL) 28-point scale score [30]. The validated CHESS score is primarily used as a risk adjustment tool to identify LTCF residents with high health instability who are likely to have adverse health outcomes, including death [31]. Medication use was defined as receiving ≥1 qualifying prescription for antipsychotics, opioid analgesics [32], antibiotics, corticosteroids, or proton pump inhibitors as well as for Beers criteria medications [33]. The Beers criteria identify specific medications and prescribing practices (e.g., excessive dose, prolonged treatment duration, harmful drug combinations, and coexisting health conditions) with evidence to suggest they should be avoided or used with caution by older adults due to unfavorable risk/benefit profiles or questionable efficacy [34]. Examples of drug classes in the Beers criteria are first generation antihistamines, barbiturates, benzodiazepines, proton-pump inhibitors, and estrogens. We measured the status of influenza vaccination for the season of cohort entry based on index date and up to date pneumococcal vaccination counting vaccinations received within or outside the LTCF. We assessed health service use as hospitalization and intensive care unit (ICU) use.

### Facility-level risk factors

As with resident-level factors, we considered facility features based on prior literature and clinical experience, including: 1) structural characteristics (urbanicity of facility location, total bed size, for-profit status); 2) staffing hours (total nursing hours/resident/day); 3) staffing type; and, 4) quality of care measures. Staffing type included proportion of registered nurses (RNs), on-site presence of a licensed independent practitioner (LIP) - either a physician assistant (PA) or an advanced practice RN (APRN), and speech language pathologist (SLP) on-staff hours per 100 beds. Quality of care measures included the percent of residents receiving antipsychotics, percent of residents restrained, and percent of residents with a pressure ulcer [19].

### Pneumonia and influenza hospitalization

We identified P&I hospitalizations by the presence of ICD-9 or ICD-10 diagnostic codes for pneumonia or influenza-like-illness (480–488.XX, J09-J18) [35, 36]. The main analysis focused on P&I diagnoses in the principal position on the claim. Secondarily, we analyzed P&I identified from any diagnosis position.

### Statistical analysis

We report the distribution of baseline characteristics of the study cohort with means and percentages for the entire cohort and among short- and long-stay residents.

The process of identifying the risk factors for P&I hospitalization proceeded in three steps. First, we grouped the variables into domains as follows: *demographics*, *admission* characteristics (location resident was admitted from, LTCF admission is new), *cardiovascular conditions* (atrial fibrillation, coronary artery disease, heart failure, hypertension, cerebrovascular accident), *respiratory conditions* (asthma/COPD/chronic lung disease, respiratory failure, pneumonia), *other medical conditions* (cancer, Parkinson’s disease, depression, diabetes mellitus, arthritis), *cognition* (Alzheimer’s and non-Alzheimer’s dementia, cognitive function scale), *physical function* (ADLs, urinary/bowel continence), *overall health stability* (CHESS scale score, Charlson comorbidity score, prognosis, prior hospitalization and/or ICU stay), *breathing* (shortness of breath, ventilator/respirator use), *eating* (tube feeding, swallowing disorders), *medication use*, *vaccinations*, and *facility* characteristics.

Second, we examined intercorrelations of variables within domains using a Pearson’s correlation coefficient matrix. None of the bivariate correlations reached a level (r > 0.8) indicating severe multicollinearity. We included state fixed effects to help account for potential state-level differences in LTCFs' propensity to hospitalize residents and code for P&I on hospital claims.

Finally, all variables from the domains identified in the first step were entered into a Cox proportional hazards model specified to account for clustering of residents within facilities using the Huber-White sandwich estimator. A stability analysis assessed an alternative Fine and Gray competing risk regression modeling approach with death as a competing outcome. Considering the large sample size, an alpha = 0.01 significance level was used to guide identification of potential P&I risk factors in the final model.

Data preparation and analyses were conducted using SAS version 9.4 (SAS Institute, Inc., Cary, NC) and Stata version 15 (StataCorp, College Station, TX). We secured administrative permission to access Medicare data through a Data Use Agreement with the Centers for Medicare and Medicaid Services (CMS). Informed consent was neither relevant nor feasible in this secondary data analysis.

## Results

### Descriptive results

#### Overall study cohort

The cohort comprised 1,711,497 individuals residing in 15,740 unique Medicare-certified LTCFs. Of these, 65.3% (*n* = 1,118,054) were short-stay and 34.7% (*n* = 593,443) long-stay residents (Additional file 1: Fig. S1). Compared to short-stay residents, long-stay residents were older (mean age: 82.9 vs. 80.8 years), and had more female (71.3% vs. 68.8%) and Black residents (11.1% vs. 7.3%) (Table 1). Generally, the prevalence of severe geriatric syndromes including cognitive impairments, dependency for ADL, and Charlson comorbidities was greater among long-stay residents than short-stay residents. Receipt of any medication in the Beers criteria was common in both short (60.1%) and long-stay (71.8%) residents. More than half received influenza (56% short-stay, 66% long-stay) and pneumococcal (67% short-stay, 72% long-stay) vaccinations. At the facility level, three-quarters of all residents were in facilities in urban areas, and nearly two-thirds (63.1%) were in facilities with at least 100 total beds.

**Table 1:** Baseline characteristics of long-term care facility residents, 2013–2015

Characteristics, n (%)	All *N* = 1,711,497	Short-stay (< 100 days) *N* = 1,118,054	Long-stay (≥100 days) *N* = 593,443
Length of follow-up, days, mean ± SD	112 ± 200	29 ± 20	269 ± 278
Age at index date, years, mean ± SD	81.6 ± 8.2	80.8 ± 8.1	82.9 ± 8.3
65–74	394,331 (23.0)	281,767 (25.2)	112,564 (19.0)
75–84	639,132 (37.3)	434,977 (38.9)	204,155 (34.4)
85+	678,034 (39.6)	401,310 (35.9)	276,724 (46.6)
Female sex	1,191,557 (69.6)	768,633 (68.8)	422,924 (71.3)
Race/ethnicity			
Non-Hispanic White	1,420,689 (83.0)	943,096 (84.4)	477,593 (80.5)
Non-Hispanic Black	147,231 (8.6)	81,401 (7.3)	65,831 (11.1)
Hispanic	62,070 (3.6)	36,682 (3.3)	25,388 (4.3)
Location resident was admitted from			
Community or home	124,588 (7.3)	37,208 (3.3)	87,380 (14.7)
Another LTCF or swing bed^a^	61,435 (3.6)	19,777 (1.8)	41,658 (7.0)
Hospital	1,486,083 (86.8)	1,044,083 (93.4)	442,000 (74.5)
LTCF admission is new	1,441,102 (84.2)	1,019,035 (91.1)	422,067 (71.1)
Body mass index, kg/m^2^			
< 18.5, underweight	117,888 (6.9)	76,489 (6.8)	41,392 (7.0)
18.5–24.9, normal	656,364 (38.4)	424,751 (38.0)	231,590 (39.0)
25–29.9, overweight	480,408 (28.0)	313,886 (28.1)	166,511 (28.1)
≥ 30, obese	456,890 (26.7)	302,928 (27.1)	153,950 (25.9)
Current tobacco use	47,959 (2.8)	24,811 (2.2)	23,148 (3.9)
Clinical Characteristics from MDS			
Cancer	141,791 (8.3)	108,447 (9.7)	33,344 (5.6)
Atrial fibrillation or other dysrhythmias	441,467 (25.8)	307,971 (27.8)	133,496 (22.5)
Coronary artery disease	452,352 (26.4)	304,647 (27.3)	147,705 (24.9)
Heart failure	385,850 (22.5)	243,464 (21.9)	142,386 (24.0)
Hypertension	1,352,541 (79.0)	877,554 (78.5)	474,987 (80.0)
History of pneumonia	125,660 (7.3)	104,590 (9.4)	21,070 (3.6)
Diabetes mellitus	554,424 (32.4)	347,402 (31.1)	207,022 (34.9)
Arthritis	517,064 (30.2)	339,561 (30.4)	177,503 (29.9)
Alzheimer’s disease	107,700 (6.3)	35,684 (3.2)	72,016 (12.1)
Cerebrovascular accident, transient ischemic attack, or stroke	197,397 (11.5)	104,438 (9.3)	92,959 (15.7)
Non-Alzheimer’s dementia	406,597 (23.8)	165,369 (14.8)	241,228 (40.7)
Depression	595,844 (34.8)	314,369 (28.1)	281,475 (47.4)
Asthma, chronic obstructive pulmonary disease, chronic lung disease	410,086 (24.0)	269,238 (24.1)	140,848 (23.7)
Respiratory failure	44,460 (2.6)	33,858 (3.0)	10,602 (1.8)
Parkinson’s disease	74,036 (4.3)	36,366 (3.3)	37,670 (6.4)
Geriatric Syndromes			
Cognitive Function Scale score			
Intact/mild cognitive impairment (0–1)	1,001,934 (58.5)	778,937 (52.1)	222,997 (37.6)
Moderate cognitive impairment (2, 3)	680,284 (39.8)	325,810 (29.1)	354,474 (59.7)
Severe cognitive impairment (4–6)	29,274 (1.7)	13,307 (1.2)	15,972 (2.7)
Activities of Daily Living 28-point Scale score			
None to limited assistance required (0–14)	795,028 (46.5)	582,547 (46.5)	212,481 (38.5)
Extensive assistance required (15–19)	620,211 (36.2)	385,371 (34.5)	234,840 (39.5)
Extensive dependency (≥20)	296,258 (17.3)	150,136 (13.4)	146,122 (24.6)
CHESS Scale score, overall health stability			
No instability (0)	990,561 (57.9)	650,661 (58.2)	339,900 (57.3)
Minimal instability (1, 2)	701,695 (41.0)	456,279 (40.8)	245,416 (41.4)
Moderate to very high instability (3+)	19,241 (1.1)	11,114 (1.0)	8127 (1.4)
Charlson comorbidity score (MDS)			
0	155,594 (9.1)	117,051 (10.5)	38,543 (6.5)
1–2	719,649 (42.1)	484,120 (43.3)	235,529 (39.7)
≥ 3	836,254 (48.9)	516,883 (46.2)	319,371 (53.8)
Urinary incontinence: frequent/always	529,756 (31.0)	238,989 (21.4)	290,767 (49.0)
Bowel incontinence: frequent/always	403,981 (23.6)	190,054 (17.0)	403,981 (23.6)
Shortness of breath	258,935 (15.1)	172,789 (15.5)	86,146 (14.5)
Swallowing disorder	65,162 (3.8)	46,058 (4.1)	19,104 (3.2)
Tube feeding	33,988 (2.0)	17,140 (1.5)	16,848 (2.8)
Ventilator or respirator use	1741 (0.1)	678 (0.1)	1063 (0.2)
Prognosis: less than 6 months to live	7145 (0.4)	5274 (0.5)	1871 (0.3)
Died during study period 2013–2015	812,036 (47.4)	459,028 (41.1)	353,008 (59.5)
Medication Use 6 months before index			
Beers criteria medication,^b^ any use	1,098,173 (64.2)	672,349 (60.1)	425,824 (71.8)
Antipsychotics, any use	96,289 (5.6)	39,659 (3.6)	56,630 (9.5)
Opioid analgesics, any use	127,139 (7.4)	99,251 (8.9)	27,888 (4.7)
Antibiotics, any use^c^	704,363 (41.2)	461,537 (41.3)	242,826 (40.9)
Corticosteroids, any use	99,079 (5.8)	72,409 (6.5)	26,670 (4.5)
Proton pump inhibitors, any use	328,345 (19.2)	229,345 (20.5)	99,000 (16.7)
Influenza vaccine received for season of cohort entry	1,021,615 (59.7)	628,356 (56.2)	393,259 (66.3)
Pneumococcal vaccination up to date	1,177,063 (68.8)	749,307 (67.0)	427,756 (72.1)
Health Service Use 6 months before index			
Any hospitalization use	1,388,076 (81.1)	1,037,211 (92.8)	350,865 (59.1)
Any ICU use	454,699 (26.6)	340,691 (30.5)	114,008 (19.2)
Facility Structural Characteristics			
Urban location	1,298,856 (75.9)	886,434 (79.3)	412,422 (69.5)
Total bed size			
< 100	630,730 (36.9)	415,999 (37.2)	214,731 (36.2)
100–200	882,091 (51.5)	570,832 (51.1)	311,259 (51.5)
> 200	198,676 (11.6)	131,223 (11.7)	67,453 (11.4)
For-profit facility	1,159,613 (67.8)	746,395 (66.8)	413,218 (69.6)
Facility Staffing Type and Hours			
Ratio of RN to RN + LPN, mean ± SD	0.4 ± 0.2	0.4 ± 0.2	0.3 ± 0.2
SLP on-staff, hours / 100 Beds, mean ± SD	0.5 ± 1.6	0.6 ± 1.6	0.4 ± 1.4
LIP on-site	773,827 (45.2)	515,034 (46.1)	258,793 (43.6)
Total nursing hours/resident/day, mean ± SD	4.9 ± 9.4	5.3 ± 10.5	4.3 ± 6.8
Facility Care Quality			
Antipsychotic use, % of residents, mean ± SD	18.9 ± 11.2	17.2 ± 10.3	22.1 ± 12.2
Restraint use, % of residents, mean ± SD	2.0 ± 4.5	1.8 ± 4.3	2.4 ± 4.8
Pressure ulcers, % of residents, mean ± SD	6.5 ± 4.7	6.7 ± 4.8	6.2 ± 4.3

*Abbreviations: LTCF* long-term care facility, *MDS* Minimum Data Set, *CHESS* Changes in Health, End-stage disease and Symptoms and Signs, *RN* Registered Nurse, *LPN* Licensed Practical Nurse, *SLP* Speech Language Pathologist, *LIP* Licensed Independent Practitioner

^a^ Swing beds are LTCF beds that can serve both short-stay and long-stay residents depending on need

^b^ The Beers criteria is a specific list of potentially inappropriate medications that are not recommended for use among older adults in most circumstances or under specific situations

^c^ Antibiotics recommended in the Infectious Diseases Society of America/American Thoracic Society consensus guidelines on the management of community-acquired pneumonia in adults

The overall prevalence of P&I hospitalizations was 3.0% for diagnoses in the principal position (short-stay 0.5%; long-stay 2.5%) and 6.6% considering any diagnosis position (short-stay 3.5%; long-stay 3.1%). At 30 days post-index, short-stay residents had 6345 hospitalizations with a principal diagnosis of P&I, and 21,942 in any diagnosis position (Table 2). Over the same period, long-stay residents had 5410 and 12,819 hospitalizations with P&I diagnoses in the principal and any position; respectively.

**Table 2:** Crude incidence rates of pneumonia and influenza-related hospitalizations among short- and long-stay residents in U.S. long-term care facilities, 2013–2015

Days since index date	Short-stay residents (N = 1,118,054)		Long-stay residents (*N* = 593,443)			
	30	90	30	90	180	365
Total person-days observed	24,395,364	32,290,058	15,668,663	41,150,984	70,681,373	111,720,666
Primary diagnosis position on the hospital claim						
Number of hospitalizations	6345	8412	5410	11,777	18,202	26,891
Crude IR per 100,000 (95% CI)	26.0 (25.4–26.6)	26.1 (25.5–26.6)	34.5 (33.6–35.4)	28.6 (28.1–29.1)	25.8 (29.4–30.2)	24.1 (23.8–24.4)
Any diagnosis position on the hospital claim						
Number of hospitalizations	21,942	28,943	12,819	28,046	43,220	63,560
Crude IR per 100,000 (95% CI)	89.9 (88.8–91.1)	89.6 (88.6–90.7)	81.8 (80.4–83.2)	68.2 (67.3–69.0)	61.1 (60.6–61.7)	56.9 (56.4–57.3)

*IR* incidence rate, *CI* confidence interval

#### Incidence rate

**Fig. 1:**
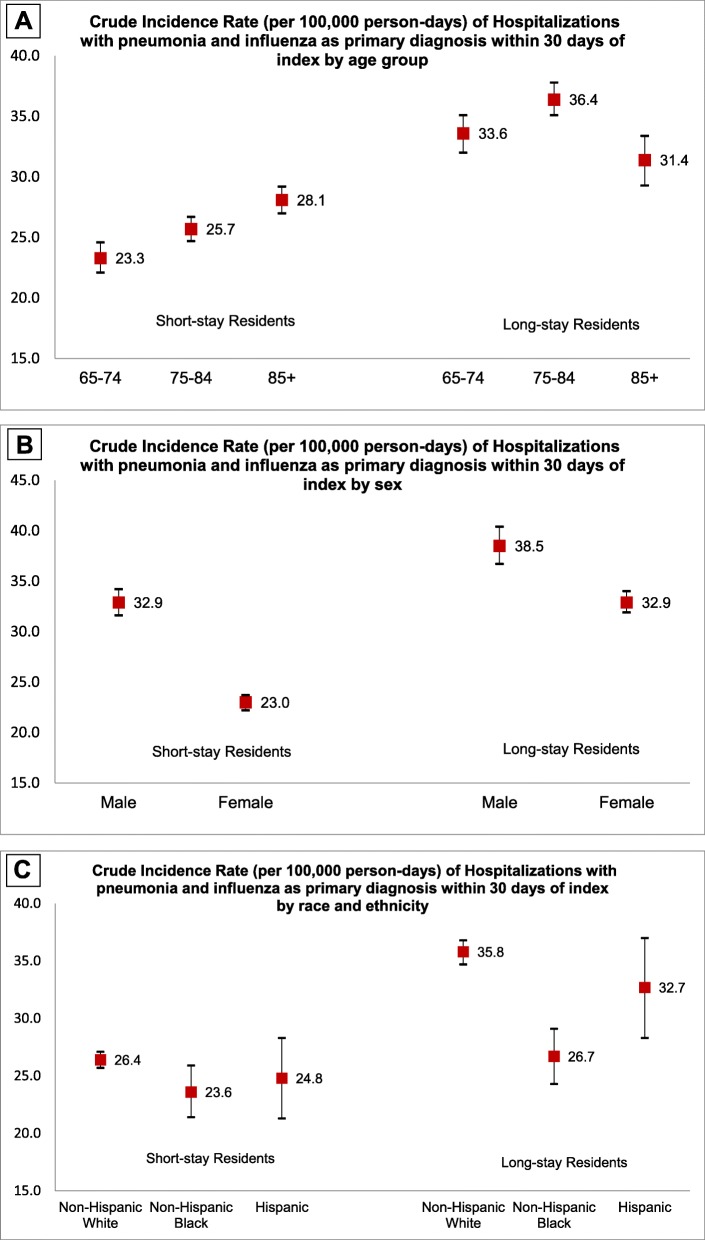
**a** Crude incidence rate (per 100,000 person-days) of hospitalizations with pneumonia and influenza as primary diagnosis within 30 days of index by age group. **b** Crude incidence rate (per 100,000 person-days) of hospitalizations with pneumonia and influenza as primary diagnosis within 30 days of index by sex. **c** Crude incidence rate (per 100,000 person-days) of hospitalizations with pneumonia and influenza as primary diagnosis within 30 days of index by race and ethnicity

For P&I hospitalizations in the principal position, the crude incidence rate (IR) and 95% confidence intervals among short-stay residents was 26.0 (25.4–26.6) per 100,000 person-days at 30 days and remained unchanged at 90 days post-index (Table 2). Among long-stay residents, the crude IR was 34.5 (33.6–35.4) at 30 days and 28.6 (28.1–29.1) at 90 days post-index. Capturing P&I in any diagnosis position yielded IRs approximately 2 to 3 times more events than using diagnoses only in the principal position. P&I incidence rates varied across age, sex, race and ethnicity (Fig. 1a-c).

### Multivariable results of cox proportional hazards models

#### Risk factors among long-stay residents

Among long-stay residents, the risk factors for P&I hospitalization included increasing age, admission from an acute care hospital, LTCF re-entry, presence of cardiovascular (atrial fibrillation, heart failure) and respiratory conditions (pneumonia, asthma/COPD, respiratory failure) in MDS assessments (Table 3). Extensive limitations in ADLs, overall health instability, and increased comorbidity burden were associated with the incidence of P&I hospitalization. Shortness of breath (HR = 1.34, 99% CI, 1.28–1.39), tube feeding (HR = 1.30, 99% CI, 1.20–1.41), and ventilator or respirator use (HR = 1.34, 99% CI, 1.03–1.75) were also identified as important risk factors. Those with any use of medications on the Beers criteria, prescriptions for antibiotics and corticosteroids, receipt of influenza or pneumococcal vaccinations, and prior ICU use had an increased rate of P&I.

**Table 3:** Results of multivariable analyses to identify factors associated with hospitalizations for P&I as the primary diagnosis among U.S. long-term care facility residents, 2013–2015

Characteristics	Short-stay^a^ HR (99% CI)^c^	Long-stay^b^ HR (99% CI)^c^
Age group (ref = 65–74)		
75–84	1.08 (1.00, 1.17)	**1.11 (1.06, 1.15)**
85+	**1.16 (1.06, 1.27)**	**1.23 (1.18, 1.28)**
Sex (ref = male)	**0.84 (0.79, 0.89)**	**0.83 (0.80, 0.85)**
Race and ethnicity		
Non-Hispanic White (ref = non-White)	1.00 (0.87, 1.15)	1.03 (0.97, 1.11)
Non-Hispanic Black (ref = non-Black)	0.86 (0.72, 1.03)	**0.89 (0.82, 0.97)**
Hispanic (ref = non-Hispanic)	1.02 (0.82, 1.27)	1.05 (0.95, 1.15)
Location resident is admitted from (ref = hospital)		
Community or home	0.81 (0.67, 0.98)	**0.79 (0.76, 0.83)**
Another LTCF or swing bed^d^	0.99 (0.77, 1.26)	**0.92 (0.87, 0.97)**
Other location	0.78 (0.60, 1.02)	**0.86 (0.81, 0.93)**
Type of admission is reentry (ref = new)	**6.50 (6.09, 6.95)**	**1.43 (1.39, 1.47)**
Body mass index, kg/m^2^ (ref = 18.5–24.9, normal)		
< 18.5, underweight	**1.14 (1.03, 1.25)**	1.05 (0.99, 1.11)
25–29.9, overweight	**0.88 (0.82, 0.95)**	0.98 (0.95, 1.01)
**≥** 30, obese	**0.74 (0.68, 0.81)**	0.98 (0.95, 1.02)
Current tobacco use (ref = no tobacco use)	0.87 (0.72, 1.06)	1.01 (0.95, 1.07)
Clinical Characteristics from MDS		
Cancer	**1.20 (1.10, 1.32)**	1.00 (0.94, 1.07)
Atrial fibrillation or other dysrhythmias	**1.13 (1.06, 1.21)**	**1.05 (1.01, 1.08)**
Coronary artery disease	1.00 (0.94, 1.07)	1.01 (0.98, 1.04)
Heart failure	1.00 (0.92, 1.08)	**1.13 (1.08, 1.18)**
Hypertension	1.02 (0.95, 1.10)	0.99 (0.96, 1.02)
History of pneumonia	**3.60 (3.36, 3.85)**	**1.38 (1.29, 1.46)**
Diabetes mellitus	0.98 (0.91, 1.05)	1.02 (0.99, 1.06)
Arthritis	**0.92 (0.85, 0.98)**	**0.95 (0.92, 0.98)**
Alzheimer’s disease	**0.79 (0.67, 0.94)**	**0.89 (0.86, 0.93)**
Cerebrovascular accident, transient ischemic attack, or stroke	**0.80 (0.72, 0.89)**	**0.89 (0.86, 0.93)**
Non-Alzheimer’s dementia	**0.91 (0.84, 0.99)**	**0.87 (0.84, 0.89)**
Depression	0.98 (0.92, 1.05)	**1.07 (1.05, 1.10)**
Asthma, chronic obstructive pulmonary disease, chronic lung disease	**1.48 (1.38, 1.58)**	**1.56 (1.51, 1.61)**
Respiratory failure	1.11 (0.99, 1.24)	**1.13 (1.03, 1.24)**
Parkinson’s disease	**0.82 (0.69, 0.97)**	0.95 (0.90, 1.00)
Geriatric Syndromes		
Cognitive Function Scale score (ref = no/mild impairment)		
Moderate cognitive impairment (2, 3)	0.95 (0.89, 1.02)	**0.90 (0.87, 0.92)**
Severe cognitive impairment (4–6)	**1.21 (1.01, 1.45)**	0.92 (0.84, 1.01)
Activities of Daily Living 28-point Scale score (ref = None to limited assistance required)		
Extensive assistance required (15–19)	**1.56 (1.45, 1.68)**	**1.08 (1.05, 1.11)**
Extensive dependency (≥20)	**1.91 (1.74, 2.08)**	**1.08 (1.04, 1.12)**
CHESS Scale score, overall health stability (ref = stable)		
Minimal instability (1, 2)	**1.21 (1.12, 1.33)**	**1.09 (1.04, 1.14)**
Moderate to very high instability (3+)	1.04 (0.84, 1.28)	**1.17 (1.04, 1.32)**
Charlson comorbidity score (MDS) (ref = 0)		
1–2	1.10 (0.96, 1.26)	1.04 (0.98, 1.10)
≥ 3	1.15 (0.99, 1.34)	**1.12 (1.04, 1.19)**
Urinary or bowel incontinence (ref = none)	**1.11 (1.04, 1.18)**	0.99 (0.96, 1.02)
Shortness of breath	**2.26 (2.09, 2.43)**	**1.34 (1.28, 1.39)**
Swallowing disorder	0.91 (0.80, 1.03)	0.96 (0.89, 1.04)
Tube feeding	0.90 (0.76, 1.07)	**1.30 (1.20, 1.41)**
Ventilator or respirator use while in facility	1.48 (0.82, 2.67)	**1.34 (1.03, 1.75)**
Prognosis: less than 6 months to live	0.88 (0.61, 1.25)	0.80 (0.62, 1.04)
*Medication Use* 6 months before index		
Beers Criteria medication,^e^ any use	**1.09 (1.03, 1.16)**	**1.09 (1.06, 1.13)**
Antipsychotics, any use	0.86 (0.74, 1.01)	**0.81 (0.77, 0.86)**
Opioid analgesics, any use	**1.11 (1.01, 1.23)**	0.97 (0.91, 1.03)
Antibiotics,^f^ any use	**1.15 (1.08, 1.22)**	**1.30 (1.27, 1.34)**
Corticosteroids, any use	1.10 (1.00, 1.22)	**1.10 (1.04, 1.17)**
Proton pump inhibitors, any use	1.04 (0.97, 1.12)	**0.87 (0.84, 0.91)**
Influenza vaccine received for season of cohort entry	0.99 (0.92, 1.05)	**1.09 (1.06, 1.13)**
Pneumococcal vaccination up to date	0.99 (0.92, 1.06)	**1.06 (1.03, 1.10)**
*Health Service Use* 6 months before index		
Any Hospitalizations	1.04 (0.92, 1.18)	**0.91 (0.88, 0.94)**
Any ICU use	1.06 (0.99, 1.13)	**1.06 (1.02, 1.11)**
Facility Structural Characteristics		
Urban location (ref = non-urban)	**0.87 (0.80, 0.93)**	**0.77 (0.74, 0.79)**
Total bed size (ref = < 100)		
100–200	0.94 (0.88, 1.01)	**0.91 (0.88, 0.94)**
> 200	0.93 (0.82, 1.06)	**0.89 (0.84, 0.94)**
For profit facility (ref = not for profit)	1.01 (0.90, 1.08)	**1.05 (1.02, 1.08)**
Facility Staffing Type and Hours		
Ratio of RN to RN + LPN (fifth vs. first quintile)	0.91 (0.81, 1.02)	**0.90 (0.85, 0.95)**
SLP on-staff hours / 100 Beds (fifth vs. first quintile)	1.02 (0.94, 1.11)	**0.99 (0.98, 1.00)**
LIP on-site (ref = none)	1.02 (0.96, 1.08)	**0.93 (0.90, 0.95)**
Total nursing hours/resident/day (fifth vs. first quintile)	**0.87 (0.78, 0.97)**	0.99 (0.95, 1.04)
Facility Care Quality		
Antipsychotic use, % of residents (fifth vs. first quintile)	1.00 (0.90, 1.10)	**1.07 (1.02, 1.11)**
Restraint use, % of residents (fifth vs. first quintile)	1.02 (0.94, 1.10)	**1.07 (1.04, 1.11)**
Pressure ulcers, % of residents (fifth vs. first quintile)	0.96 (0.87, 1.05)	1.01 (0.96, 1.05)

Values in boldface indicate statistically significant associations at the 0.01 level

*Abbreviations: LTCF* long-term care facility, *MDS* Minimum Data Set, *CHESS* Changes in Health, End-stage disease and Symptoms and Signs, *RN* Registered Nurse, *LPN* Licensed Practical Nurse, *SLP* Speech Language Pathologist, *LIP* Licensed Independent Practitioner

^a^ *N* = 1,080,816 after 37,238 (3.3%) were excluded from regression due to missing data on facility-level variables

^b^ *N* = 571,694 after 21,749 (3.7%) were excluded from regression due to missing data on facility-level variables

^c^ We used robust Huber-White standard errors to account for clustering of residents within LTCFs

^d^ Swing beds are LTCF beds that can serve both short-stay and long-stay residents depending on need

^e^ The Beers criteria is a specific list of potentially inappropriate medications that are not recommended for use among older adults in most circumstances or under specific situations

^f^ Antibiotics recommended in the Infectious Diseases Society of America/American Thoracic Society consensus guidelines on the management of community-acquired pneumonia in adults

P&I hospitalizations were less common among residents who were female, Black, diagnosed with certain conditions such as dementia, and prescribed antipsychotics than among residents without these characteristics.

Facility-level characteristics associated with higher risk of P&I hospitalization among long-stay residents were for-profit status and poor care quality measures such as greater use of antipsychotics or restraints. Residents in LTCFs with more RNs, and having LIPs and SLPs on site had a decreased rate of P&I compared with those at facilities without such staffing. LTCFs located in urban settings (HR = 0.77, 99% CI, 0.74–0.79) and with higher bed capacity (HR = 0.89, 99% CI, 0.84–0.94 for > 200 beds versus < 200 beds) were associated with lower P&I hospitalizations.

#### Risk factors among short-stay residents

Results among the short-stay population were largely consistent with the direction of findings in the long-stay population although the magnitude of the associations tended to differ. Receipt of influenza and pneumococcal vaccination suggested a 1% decreased rate of P&I hospitalization among short-stay residents; however, this association did not meet statistical significance. While facility structural characteristics, staffing type, and care quality measures were important predictors among long-stay residents; these variables were not associated with P&I among short-stay residents.

#### Secondary analyses

Considering P&I diagnoses in any position, rather than those only in the primary position, indicated variation in how the variables were related to P&I hospitalization (Additional file 1: Table S1). For example, having received influenza vaccination (HR = 0.97, 99% CI, 0.94–0.99) was protective against P&I hospitalization among short-stay residents when P&I diagnoses in any position were considered in the outcome. The competing risk analyses yielded interpretations substantively similar to the main results; however, select medical conditions (e.g., cancer, atrial fibrillation) had inconsistent findings (Additional file 1: Table S2).

## Discussion

This study examined risk factors for P&I hospitalization among short- and long-stay residents of LTCFs. The incidence of P&I hospitalizations varied between these cohorts. Despite several shared risk factors, there also were differences in the direction and magnitude of the associations across short and long-stay residents depending on whether P&I diagnoses in the principal versus any diagnosis position were considered, and based on accounting for death as a competing risk.

In the main analysis focused on hospitalizations with P&I in the principal position, resident-level variables that were consistently associated with increased risk among both short and long-stay residents were older age (85+), admission from an acute hospital, LTCF reentry, atrial fibrillation/dysrhythmias, asthma/COPD/chronic lung disease, extensive ADL limitations, shortness of breath, Beers criteria medication use, and history of antibiotic prescriptions. Among facility characteristics, urban location was associated with lower rates of P&I hospitalizations for both short and long-stay residents. We observed that being overweight or obese was associated with reduced risk for P&I hospitalization among short-stay residents. This may reflect the obesity paradox [37]; however, the underlying mechanism for this likely non-linear relationship remains unclear and has been reported for pneumonia in reduction of mortality rather than hospitalization [38].

The variations we observed in the direction and/or magnitude of the hazard ratios depending on resident type (short vs. long-stay) and P&I diagnosis position (principal vs. any) call attention to the need for nuanced strategies for preventing and controlling P&I considering these patient profiles. In particular, potentially modifiable risk factors at both individual (e.g., medication use, chronic disease management of cardiorespiratory conditions) and facility levels (e.g., staffing, care quality) represent important opportunities to reduce the incidence of P&I-related hospitalizations. We acknowledge the unexpected results whereby select conditions (e.g., Parkinson’s disease, dementia, arthritis) and individual-level medication use (e.g., antipsychotic, proton pump inhibitors) appeared to confer protective effects among short- and/or long-stay residents. This represents an opportunity for future research into these associations.

The positive relationship between vaccinations and P&I hospitalizations among long-stay residents could reflect a tendency for those at greatest risk of P&I infection or most vulnerable to hospitalization to receive vaccines. If so, vaccine use in LTCF residents would be subject to confounding by indication. Research that assesses provider and patient decision-making regarding the offer and acceptance of vaccination may shed additional light on this. Nonetheless, annual influenza vaccination is recommended and has been found cost-effective in preventing and controlling infection and other negative sequelae [39]. There is uncertainty about the effectiveness of the pneumococcal vaccine at preventing pneumonia in the elderly [40–42]; however, limited evidence suggests the vaccine may decrease in-hospital death, length of hospital stay and the need for ICU admission among those with community-acquired pneumonia [43, 44]. As such, there is benefit in recommending pneumococcal vaccination and broadly increasing vaccination coverage in LTCFs [45]. Furthermore, improving pneumococcal vaccination rates and preventing pneumonia is a priority for CMS. For example, the agency promulgated immunization standards as part of the LTCF conditions of participation [46], and initiated public reporting of pneumococcal vaccination rates [47]. Additionally, 30-day mortality and hospital readmission measures for pneumonia are part of the hospital quality initiative [48]. Our results identifying individuals at highest risk for P&I can be used to target prevention interventions to those residents who are most likely to benefit, and thus may help providers comply with CMS quality initiatives.

This study has limitations. First, our definition of P&I hospitalization relies on inpatient claims alone and misses infections that are not evaluated by diagnostic testing or those that did not result in hospitalization. Furthermore, P&I may be underestimated due to coding practices that arise when providers confer higher priority to other comorbidities over P&I for billing purposes, or if P&I go unrecognized due to the severity of co-occurring clinical conditions. Nonetheless, our sensitivity analysis capturing P&I diagnoses in all positions on the claim help address this limitation. Second, information on risk factors was ascertained potentially at least 6 months before the onset of the outcome in long-stay residents. Therefore, resident characteristics at the time of P&I may not have fully aligned with those present at baseline. However, our approach maintained the temporal relationship between risk factors and the outcome. Third, relative to long-stay residents, short-stay residents more frequently had missing information on MDS-derived variables (and thus more likely to be excluded from analysis) as they may not have had as many MDS assessments. By excluding residents with missing information, we avoided making untenable assumptions that would be required to conduct multiple imputation and related approaches to handle missing data. Fourth, direct observation is longer for long-stay than short stay residents, as the risk exposure time is truncated upon short stay residents’ discharge to the community. This potentially underestimates the risk of P&I hospitalization among short-stay residents though our focus was on events occurring within LTCFs. Furthermore, to the extent that LTCFs have different tendencies to transfer short versus long stay residents to the hospital, the observed IRs for P&I could be differentially underestimated. Finally, the study population comprised Medicare beneficiaries residing in LTCFs and were ≥ 65 years of age; therefore, our findings may not extend to other settings (e.g., community), younger LTCF residents, or beneficiaries of other insurance sources.

## Conclusions

In an older adult LTCF population, IRs of P&I hospitalizations were greater among long-stay than short-stay residents. There were variations in the risk factors, and magnitude of associations, that predicted P&I hospitalizations between short- and long-stay residents when using principal versus any P&I diagnosis position and differing modeling approaches. Nonetheless, we identified several risk factors in common in these two populations including advanced age, admission from an acute hospital, presence of cardiovascular and respiratory conditions, impaired functional status, receipt of antibiotics, and prescriptions meeting the Beers criteria for potentially inappropriate use in older adults. Interventions aimed at improving infection prevention and control in LTCFs should be differentiated depending on resident type as those primarily receiving limited duration post-acute care may require different strategies than longer term residents.

## Supplementary information

**Additional file 1: Figure S1.** Sample selection flow diagram. **Table S1.** Multivariable analysis of factors associated with hospitalizations for P&I in any diagnosis position. **Table S2.** Hazard ratios based on Fine and Gray competing risk analyses accounting for death among hospitalizations with P&I as the principal diagnosis.

## Data Availability

We are unable to provide the study data in accordance with our data use agreement with the Centers for Medicare and Medicaid Services.

## Abbreviations

ADL: Activities of Daily Living
APRN: Advanced Practice Registered Nurse
CHESS: Changes in Health, End-stage disease and Symptoms and Signs
CI: Confidence Interval
CMS: Centers for Medicare and Medicaid Services
COPD: Chronic Obstructive Pulmonary Disease
HR: Hazard Ratio
ICD-10: International Classification of Diseases, Tenth Revision
ICD-9: International Classification of Diseases, Ninth Revision
ICU: Intensive Care Unit
LIP: Licensed Independent Practitioner
LTCF: Long-Term Care Facility
MDS: Minimum Data Set
OSCAR/CASPER: Online Survey and Certification And Survey Provider Enhanced Reports
P&I: Pneumonia and Influenza
PA: Physician Assistant
RN: Registered Nurse
SLP: Speech Language Pathologist
