# Supplementary material for: Risk factors for pneumonia and influenza hospitalizations in long-term care facility residents: a retrospective cohort study
Source: BMC Geriatr. 2020 Feb 10;20:47. doi: 10.1186/s12877-020-1457-8 (PMC7011520; doi:10.1186/s12877-020-1457-8)
Supplement: Supplementary file 1 — Additional file 1: Figure S1. Sample selection flow diagram. Table S1. Multivariable analysis of factors associated with hospitalizations for P&I in any diagnosis position. Table S2. Hazard ratios based on Fine and Gray competing risk analyses accounting for death among hospitalizations with P&I as the principal diagnosis. [file 12877_2020_1457_MOESM1_ESM.docx]

**SUPPLEMENTAL ONLINE CONTENT**

**Figure S1**: Sample selection flow diagram

**Table S1:** Multivariable analysis of factors associated with hospitalizations for P&I in any diagnosis position

**Table S2:** Hazard ratios based on Fine and Gray competing risk analyses accounting for death among hospitalizations with P&I as the principal diagnosis

**Figure S1**: Sample selection flow diagram

Excluded:

- Medicare Advantage enrolled,

n= 1,842,815

- No Medicare Part D 6 months before index,

n= 1,366,673

- Hospice stay,

n= 57,962

Medicare beneficiaries aged ≥65 years in long-term care facilities during 2013-2015

N = 5, 772, 762

N = 1,711,497

N = 2,505,312

Long-stay residents

N = 593, 443

Short-stay residents

N = 1, 118, 054

Excluded:

- Missing data on key variables, n= 793,815

**TABLE S1.** Multivariable analysis of factors associated with hospitalizations for P&I in any diagnosis position

| Characteristics | Short-stay^a^  HR (99% CI) | Long-stay^b^  HR (99% CI) |
| --- | --- | --- |
| Age group (ref = 65-74) |  |  |
| 75-84 | **1.07 (1.04, 1.10)** | 1.02 (0.99, 1.06) |
| 85+ | **1.11 (1.08, 1.15)** | **1.06 (1.02, 1.09)** |
| Sex (ref = male) | **0.84 (0.82, 0.86)** | **0.82 (0.80, 0.84)** |
| Race and ethnicity |  |  |
| Non-Hispanic White (ref = non-White) | 0.98 (0.93, 1.04) | 1.00 (0.94, 1.06) |
| Non-Hispanic Black (ref = non-Black) | 0.95 (0.89, 1.02) | **0.92 (0.86, 0.99)** |
| Hispanic (ref = non-Hispanic) | **1.18 (1.09, 1.27)** | **1.10 (1.01, 1.19)** |
| Location resident is admitted from (ref = acute hospital) |  |  |
| Community or home | **0.83 (0.78, 0.89)** | **0.80 (0.77, 0.83)** |
| Another LTCF or swing bed^c^ | 1.08 (0.99, 1.17) | **0.90 (0.86, 0.94)** |
| Other location | **0.82 (0.74, 0.90)** | **0.91 (0.86, 0.97)** |
| Type of admission is reentry (ref = new) | **2.39 (2.32, 2.46)** | **1.40 (1.37, 1.44)** |
| Body mass index, kg/m^2^ (ref = 18.5-24.9, normal) |  |  |
| <18.5, underweight | **1.08 (1.04, 1.13)** | 1.04 (0.99, 1.09) |
| 25 – 29.9, overweight | **0.94 (0.92, 0.97)** | 0.99 (0.96, 1.01) |
| ≥30, obese | **0.86 (0.84, 0.89)** | 0.99 (0.96, 1.02) |
| Current tobacco use (ref = no tobacco use) | 1.00 (0.93, 1.07) | 1.02 (0.97, 1.08) |
| *Clinical Characteristics from MDS* |  |  |
| Cancer | **1.01 (0.97, 1.05)** | 1.01 (0.95, 1.06) |
| Atrial fibrillation or other dysrhythmias | **1.09 (1.06, 1.12)** | **1.05 (1.02, 1.08)** |
| Coronary artery disease | **1.06 (1.04, 1.09)** | 1.02 (0.99, 1.05) |
| Heart failure | 1.04 (1.00, 1.07) | **1.11 (1.07, 1.15)** |
| Hypertension | 0.98 (0.96, 1.01) | 0.99 (0.96, 1.02) |
| Pneumonia | **2.40 (2.34, 2.47)** | **1.38 (1.31, 1.46)** |
| Diabetes mellitus | 1.03 (1.00, 1.06) | 1.03 (1.00, 1.09) |
| Arthritis | **0.92 (0.89, 0.94)** | **0.96 (0.93, 0.98)** |
| Alzheimer’s disease | **0.88 (0.82, 0.94)** | **0.87 (0.84, 0.88)** |
| Cerebrovascular accident, transient ischemic attack, or stroke | **0.92 (0.89, 0.96)** | **0.88 (0.85, 0.91)** |
| Non-Alzheimer’s dementia | **0.93 (0.90, 0.96)** | **0.84 (0.82, 0.86)** |
| Depression | **1.04 (1.02, 1.07)** | **1.06 (1.04, 1.09)** |
| Asthma, chronic obstructive pulmonary disease, chronic lung disease | **1.61 (1.57, 1.65)** | **1.56 (1.51, 1.60)** |
| Respiratory failure | **1.08 (1.03, 1.14)** | **1.18 (1.09, 1.28)** |
| Parkinson’s disease | **0.97 (0.91, 1.03)** | **0.95 (0.90, 0.99)** |
| *Geriatric Syndromes* |  |  |
| Cognitive Function Scale score (ref = no/mild impairment) |  |  |
| Moderate cognitive impairment (2-3) | **0.92 (0.90, 0.95)** | **0.86 (0.84, 0.88)** |
| Severe cognitive impairment (4-6) | 0.99 (0.90, 1.09) | **0.85 (0.79, 0.93)** |
| Activities of Daily Living 28-point Scale score (ref = None to limited assistance required) |  |  |
| Extensive assistance required (15-19) | **0.93 (0.91, 0.95)** | **1.06 (1.03, 1.09)** |
| Extensive dependency (≥20) | **0.85 (0.82, 0.88)** | 1.03 (1.00, 1.07) |
| CHESS Scale score, overall health stability (ref = stable) |  |  |
| Minimal instability (1-2) | **1.17 (1.13, 1.21)** | **1.09 (1.04, 1.13)** |
| Moderate to very high instability (3+) | 1.09 (0.99, 1.21) | **1.16 (1.03, 1.32)** |
| Charlson comorbidity score (MDS) (ref = 0) |  |  |
| 1-2 | **1.08 (1.03, 1.14)** | 1.05 (1.00, 1.11) |
| ≥3 | **1.16 (1.09, 1.22)** | **1.14 (1.07, 1.21)** |
| Urinary or bowel incontinence (ref = none) | 0.99 (0.96, 1.01) | 0.99 (0.96, 1.01) |
| Shortness of breath | **1.37 (1.33, 1.41)** | **1.30 (1.25, 1.34)** |
| Swallowing disorder | **0.88 (0.84, 0.93)** | 0.95 (0.89, 1.02) |
| Tube feeding | 1.06 (0.98, 1.15) | **1.39 (1.29, 1.48)** |
| Ventilator or respirator use | 0.97 (0.69, 1.36) | **1.29 (1.03, 1.60)** |
| Prognosis: less than 6 months to live | **0.66 (0.55, 0.79)** | **0.71 (0.56, 0.91)** |
| *Medication Use* 6 months before index |  |  |
| Beers Criteria medication,^d^ any use | **1.17 (1.14, 1.20)** | **1.10 (1.07, 1.13)** |
| Antipsychotics, any use | **0.92 (0.87, 0.98)** | **0.82 (0.79, 0.86)** |
| Opioid analgesics, any use | **1.21 (1.17, 1.25)** | 1.00 (0.94, 1.05) |
| Antibiotics,^e^ any use | **1.34 (1.31, 1.37)** | **1.27 (1.24, 1.30)** |
| Corticosteroids, any use | **1.18 (1.14, 1.23)** | **1.07 (1.01, 1.13)** |
| Proton pump inhibitors, any use | **1.12 (1.09, 1.15)** | **0.87 (0.84, 0.90)** |
| Influenza vaccine received for season of cohort entry | **0.97 (0.94, 0.99)** | **1.06 (1.02, 1.08)** |
| Pneumococcal vaccination up to date | **1.09 (1.06, 1.12)** | **1.05 (1.02, 1.10)** |
| *Health Service Use* 6 months before index |  |  |
| Any Hospitalizations | **1.37 (1.30, 1.44)** | **1.03 (1.00, 1.05)** |
| Any ICU use | **1.11 (1.08, 1.13)** | **1.13 (1.09, 1.17)** |
| Facility Structural Characteristics |  |  |
| Urban location (ref = non-urban) | **0.96 (0.93, 0.98)** | **0.80 (0.78, 0.82)** |
| Total bed size (ref = <100) |  |  |
| 100-200 | 0.98 (0.94, 1.01) | **0.92 (0.90, 0.94)** |
| >200 | **0.93 (0.88, 0.97)** | **0.90 (0.86, 0.94)** |
| For profit facility (ref = not for profit) | **0.96 (0.93, 0.98)** | **1.08 (1.05, 1.11)** |
| Facility Staffing Type and Hours |  |  |
| Ratio of RN to RN+LPN (fifth vs. first quintile) | **1.07 (1.03, 1.12)** | 1.01 (0.98, 1.05) |
| SLP on-staff hours / 100 Beds (fifth vs. first quintile) | 1.05 (0.99, 1.11) | 0.99 (0.96, 1.03) |
| LIP on-site (ref = none) | 1.00 (0.98, 1.02) | **0.94 (0.92, 0.96)** |
| Total nursing hours/resident/day (fifth vs. first quintile) | **1.14 (1.09, 1.18)** | 1.01 (0.97, 1.05) |
| Facility Care Quality |  |  |
| Antipsychotic use, % of residents (fifth vs. first quintile) | **0.94 (0.90, 0.97)** | **1.07 (1.03, 1.11)** |
| Restraint use, % of residents (fifth vs. first quintile) | **0.96 (0.93, 0.99)** | **1.05 (1.02, 1.09)** |
| Pressure ulcers, % of residents (fifth vs. first quintile) | **1.07 (1.03, 1.12)** | **1.05 (1.01, 1.09)** |

**Abbreviations:** LTCF, long-term care facility; MDS, Minimum Data Set; CHESS, Changes in Health, End-stage disease and Symptoms and Signs; RN, Registered Nurse; LPN, Licensed Practical Nurse; SLP, Speech Language Pathologist; LIP, Licensed Independent Practitioner.

^a^ N=1,080,816 after 37,238 (3.3%) were excluded from regression due to missing data on facility-level variables. Values in boldface indicate statistically significant associations at the 0.01 level.

^b^ N=571,694 after 21, 749 (3.7%) were excluded from regression due to missing data on facility-level variables. Values in boldface indicate statistically significant associations at the 0.01 level.

^c^ Swing beds are LTCF beds that can serve both short-stay and long-stay residents depending on need

^d^ The Beers criteria is a specific list of potentially inappropriate medications that are not recommended for use among older adults in most circumstances or under specific situations

^e^ Antibiotics recommended in the Infectious Diseases Society of America/American Thoracic Society consensus guidelines on the management of community-acquired pneumonia in adults.

**Table S2:** Hazard ratios based on Fine and Gray competing risk analyses accounting for death among hospitalizations with P&I as the principal diagnosis ^a^

| Characteristics | Short-stay^b^  HR (99% CI) | Long-stay^c^  HR (99% CI) |
| --- | --- | --- |
| Age group (ref = 65-74) |  |  |
| 75-84 | **1.10 (1.01, 1.19)** | **1.12 (1.08, 1.16)** |
| 85+ | **1.19 (1.09, 1.29)** | **1.25 (1.21, 1.29)** |
| Sex (ref = male) | **0.88 (0.83, 0.94)** | **0.91 (0.88, 0.94)** |
| Race and ethnicity |  |  |
| White (ref = non-White) | 1.04 (0.90, 1.19) | 1.00 (0.93, 1.07) |
| Black (ref = non-Black) | 0.91 (0.77, 1.09) | **0.90 (0.82, 0.98)** |
| Hispanic (ref = non-Hispanic) | 1.04 (0.84, 1.28) | 1.06 (0.96, 1.15) |
| Location resident is admitted from (ref = hospital) |  |  |
| Community or home | 0.89 (0.73, 1.07) | **0.78 (0.74, 0.82)** |
| Another LTCF or swing bed^d^ | 1.02 (0.81, 1.29) | **0.98 (0.93, 1.03)** |
| Other location | 0.88 (0.68, 1.15) | **0.91 (0.84, 0.98)** |
| Type of admission is reentry (ref = new) | **5.36 (5.02, 5.72)** | **1.41 (1.38, 1.44)** |
| Body mass index (ref = 18.5-24.9, normal) |  |  |
| <18.5, underweight | **1.13 (1.02, 1.24)** | **0.94 (0.89, 0.99)** |
| 25 – 29.9, overweight | **0.93 (0.84, 0.97)** | **1.05 (1.01, 1.08)** |
| ≥30, obese | **0.79 (0.73, 0.86)** | **1.10 (1.06, 1.14)** |
| Current tobacco use (ref = no tobacco use) | 0.90 (0.74, 1.08) | **1.08 (1.02, 1.14)** |
| *Clinical Characteristics from MDS* |  |  |
| Cancer | **1.11 (1.01, 1.21)** | **0.85 (0.79, 0.91)** |
| Atrial fibrillation or other dysrhythmias | **1.10 (1.03, 1.17)** | **0.96 (0.93, 0.99)** |
| Coronary artery disease | 0.99 (0.93, 1.06) | 0.96 (0.93, 1.00) |
| Heart failure | 0.96 (0.89, 1.03) | 1.04 (1.00, 1.08) |
| Hypertension | 1.03 (0.96, 1.11) | 0.99 (0.96, 1.03) |
| Pneumonia | **3.56 (3.14, 3.59)** | **1.24 (1.18, 1.30)** |
| Diabetes mellitus | 0.97 (0.91, 1.04) | 0.97 (0.94, 1.00) |
| Arthritis | 0.96 (0.91, 1.04) | 0.99 (0.96, 1.01) |
| Alzheimer’s disease | **0.83 (0.70, 0.98)** | **0.94 (0.90, 0.98)** |
| Cerebrovascular accident, transient ischemic attack, or stroke | **0.84 (0.76, 0.93)** | 0.96 (0.93, 1.00) |
| Non-Alzheimer’s dementia | 0.94 (0.87, 1.03) | **0.90 (0.87, 0.93)** |
| Depression | 1.02 (0.96, 1.09) | **1.11 (1.09, 1.14)** |
| Asthma, chronic obstructive pulmonary disease, chronic lung disease | **1.43 (1.34, 1.54)** | **1.46 (1.43, 1.49)** |
| Respiratory failure | 1.05 (0.94, 1.18) | 0.99 (0.89, 1.08) |
| Parkinson’s disease | 0.87 (0.74, 1.03) | 0.99 (0.94, 1.05) |
| *Geriatric Syndromes* |  |  |
| Cognitive Function Scale score (ref = no/mild impairment) |  |  |
| Moderate cognitive impairment (2-3) | 0.95 (0.88, 1.01) | **0.88 (0.85, 0.91)** |
| Severe cognitive impairment (4-6) | 1.16 (0.97, 1.39) | **0.84 (0.75, 0.93)** |
| Activities of Daily Living 28-point Scale score (ref = None to limited assistance required) |  |  |
| Extensive assistance required (15-19) | **1.49 (1.39, 1.60)** | **0.94 (0.91, 0.97)** |
| Extensive dependency (≥20) | **1.71 (1.56, 1.87)** | **0.90 (0.86, 0.93)** |
| CHESS Scale score, overall health stability (ref = stable) |  |  |
| Minimal instability (1-2) | **1.19 (1.09, 1.29)** | **1.05 (1.01, 1.10)** |
| Moderate to very high instability (3+) | 1.02 (0.83, 1.25) | **1.09 (0.97, 1.21)** |
| Charlson comorbidity score (MDS) (ref = 0) |  |  |
| 1-2 | 1.07 (0.93, 1.22) | 1.01 (0.95, 1.07) |
| ≥3 | 1.09 (0.93, 1.27) | 1.05 (0.99, 1.12) |
| Urinary or bowel incontinence (ref = none) | **1.13 (1.06, 1.20)** | 0.98 (0.96, 1.01) |
| Shortness of breath | **2.09 (1.94, 2.25)** | **1.25 (1.21, 1.28)** |
| Swallowing disorder | 0.89 (0.79, 1.01) | 0.94 (0.87, 1.02) |
| Tube feeding | 0.94 (0.80, 1.11) | **1.24 (1.16, 1.32)** |
| Ventilator or respirator use | 1.51 (0.85, 2.70) | **1.40 (1.13, 1.66)** |
| Prognosis: less than 6 months to live | 0.77 (0.54, 1.09) | **0.73 (0.47, 0.98)** |
| *Medication Use* 6 months before index |  |  |
| Had Beers Criteria medication^e^ | **1.07 (1.01, 1.14)** | **1.09 (1.06, 1.12)** |
| Any use of antipsychotics | 0.91 (0.77, 1.06) | **0.81 (0.76, 0.86)** |
| Any use of opioid analgesics | 1.09 (0.99, 1.21) | 0.96 (0.90, 1.03) |
| Any use of select antibiotics^f^ | **1.11 (1.05, 1.18)** | **1.27 (1.24, 1.29)** |
| Any use of corticosteroids | 1.09 (0.99, 1.20) | 1.06 (1.00, 1.12) |
| Any use of proton pump inhibitors | 1.03 (0.96, 1.10) | **0.86 (0.82, 0.89)** |
| Influenza vaccine received for season of cohort entry | 1.01 (0.95, 1.08) | **1.13 (1.10, 1.16)** |
| Pneumococcal vaccination up to date | 0.98 (0.91, 1.05) | **1.07 (1.04, 1.10)** |
| *Health Service Use* 6 months before index |  |  |
| Any Hospitalizations | 1.02 (0.90, 1.15) | **0.74 (0.71, 0.77)** |
| Any ICU use | 1.03 (0.97, 1.09) | **0.95 (0.91, 0.99)** |
| Facility Structural Characteristics |  |  |
| Urban location (ref = non-urban) | **0.79 (0.73, 0.84)** | **0.70 (0.67, 0.73)** |
| Total bed size (ref = <100) |  |  |
| 100-200 | 0.95 (0.89, 1.01) | **0.89 (0.87, 0.92)** |
| >200 | 0.96 (0.85, 1.07) | **0.86 (0.81, 0.91)** |
| For profit facility (ref = not for profit) | 1.00 (0.94, 1.07) | 1.03 (1.00, 1.06) |
| Facility Staffing Type and Hours |  |  |
| Ratio of RN to RN+LPN (fifth vs. first quintile) | **0.86 (0.78, 0.95)** | **0.83 (0.79, 0.87)** |
| SLP on-staff hours / 100 Beds (fifth vs. first quintile) | 0.99 (0.91, 1.07) | **0.92 (0.88, 0.95)** |
| LIP on-site (ref = none) | 1.01 (0.95, 1.07) | **0.91 (0.88, 0.94)** |
| Total nursing hours/resident/day (fifth vs. first quintile) | **0.79 (0.71, 0.87)** | **0.90 (0.86, 0.94)** |
| Facility Care Quality |  |  |
| Antipsychotic use, % of residents (fifth vs. first quintile) | 1.04 (0.94, 1.16) | **1.12 (1.07, 1.16)** |
| Restraint use, % of residents (fifth vs. first quintile) | 1.03 (0.95, 1.10) | **1.08 (1.04, 1.11)** |
| Pressure ulcers, % of residents (fifth vs. first quintile) | 0.96 (0.87, 1.05) | 0.98 (0.93, 1.02) |

**Abbreviations:** LTCF, long-term care facility; MDS, Minimum Data Set; CHESS, Changes in Health, End-stage disease and Symptoms and Signs; RN, Registered Nurse; LPN, Licensed Practical Nurse; SLP, Speech Language Pathologist; LIP, Licensed Independent Practitioner.

^a^ Modeling hospitalizations with pneumonia and influenza as the principal diagnosis. We dropped the state fixed effect in the competing risk model in order to resolve convergence issues.

^b^ N=1,080,816 after 37,238 (3.3%) were excluded from regression due to missing data on facility-level variables. Values in boldface indicate statistically significant associations at the 0.01 level.

^c^ N=571,694 after 21, 749 (3.7%) were excluded from regression due to missing data on facility-level variables. Values in boldface indicate statistically significant associations at the 0.01 level.

^d^ Swing beds are LTCF beds that can serve both short-stay and long-stay residents depending on need

^e^ The Beers criteria is a specific list of potentially inappropriate medications that are not recommended for use among older adults in most circumstances or under specific situations

^f^ Antibiotics recommended in the Infectious Diseases Society of America/American Thoracic Society consensus guidelines on the management of community-acquired pneumonia in adults.
